# Biophysical properties of a tau seed

**DOI:** 10.1038/s41598-021-93093-z

**Published:** 2021-06-30

**Authors:** Zhiqiang Hou, Dailu Chen, Bryan D. Ryder, Lukasz A. Joachimiak

**Affiliations:** 1 Center for Alzheimer’s and Neurodegenerative Diseases, University of Texas Southwestern Medical Center, Dallas, TX 75390 USA; 2 Molecular Biophysics Graduate Program, University of Texas Southwestern Medical Center, Dallas, TX 75390 USA; 3 Department of Biochemistry, University of Texas Southwestern Medical Center, Dallas, TX 75390 USA

**Keywords:** Prions, Molecular conformation, Supramolecular assembly

## Abstract

Pathogenesis of tauopathies involves conversion of tau monomer into pathological tau conformers that serve as templates to recruit native tau into growing assemblies. Small soluble tau seeds have been proposed to drive pathological tau assembly in vitro, in cells and in vivo. We have previously described the isolation of monomeric pathogenic tau seeds derived from recombinant samples and tauopathy tissues but in-depth biophysical characterization of these species has not been done. Here we describe a chromatographic method to isolate recombinant soluble tau seeds derived from heparin treatment. We used biochemical and biophysical approaches to show that the seeds are predominantly monomeric and have the capacity to nucleate aggregation of inert forms of tau in vitro and in cells. Finally, we used crosslinking mass spectrometry to identify the topological changes in tau as it converts from an inert state to a pathogenic seed. Future studies will reveal the relationship between soluble seeds and structural polymorphs derived from tauopathies to help diagnose and develop therapeutics targeting specific tauopathies.

## Introduction

Under normal physiologic conditions, tau is stable and does not readily aggregate in the absence of inducers^1^. Early analyses of tau structure suggested that it does not adopt a folded conformation, but rather is intrinsically disordered^2^. Given that tau encodes amyloid motifs that mediate self-assembly^3^, a key question is what limits the self-association of amyloid motifs to yield aggregation-resistant tau under normal physiologic conditions? Recent isolation and characterization of distinct pools of tau monomer, some with properties of seeding and self-assembly, and others without, indicates that tau adopts a structure surrounding the amyloid motifs that regulates aggregation^4^.

Prior studies have proposed that repeat domains encode local structure^5^. Tau has been shown to adopt structure surrounding the PGGG motifs, at the end of each repeat, that precede amyloid motifs^6^. Disease-associated mutations are enriched upstream of the amyloid motifs^7^. We have characterized the sequences surrounding the ^306^VQIVYK^311^ amyloid motif, finding that, disease-associated mutations upstream from this motif promote aggregation of tau by disrupting the protective local structures^3^. Thus, the formation of these protective structures limits aggregation but is still compatible with conformations required for association with microtubules. A model based on local structures that mask aggregation properties could explain tau’s stability and inability to aggregate in the absence of inducers. Interestingly, similar concepts have emerged for other intrinsically disordered proteins encoding amyloid motifs such as α-synuclein, where design of different β-turn types modulates aggregation properties^8^.

Initiation of tau aggregation in vitro requires the addition of preformed tau seeds or incubation with polyanions, such as heparin, disrupt these aggregation-protective structures^9,10^. Heparin binds to the repeat 2 in the repeat domain of tau, stabilizing it in an unfolded conformation^11–13^. Polyanion binding to positively charged residues in the repeat domain may preferentially expose sequences that promote oligomer assembly during the lag phase followed by the elongation adhering to a classical nucleation mechanism^14^. In prior work, we isolated distinct pools of tau monomer, from both recombinant and brain-derived sources, indicating that tau may exist in distinct separable conformations with different aggregation properties. Tau monomer that is otherwise aggregation-resistant has the capacity to adopt aggregation-prone conformations that self-assemble and initiate aggregation in vitro and in the setting of disease states^4^.

Structural and modeling analyses comparing inert and seed-competent tau monomers revealed preferential exposure of amyloid motifs (^275^VQIINK^280^ and ^306^VQIVYK^311^) in seed-competent tau monomer which then can act as a nucleus to promote elongation^4^. Furthermore, the seed-competent form of tau monomer isolated from distinct tauopathies has been observed to encode distinct subsets of strains^15^. This indicates a possible ensemble of aggregation-prone monomeric conformers that have the capacity to adopt and propagate distinct fibrillar conformations^15^. The idea that tau monomer alone can drive its assembly and serve as a template to form structural polymorphs is not widely accepted, although recent work from other groups on tau^16^, and Sup35^17^, are consistent with this idea.

Here we describe a chromatographic approach to produce monomeric heparin-induced tau seeds, herein referred to as M_s_. We used cell-biological and biochemical approaches to study the properties of the M_s_ in vitro and in cells revealing that nanomolar amounts of seeds can trigger aggregation of inert tau (herein referred to as M_i_). We also show using biophysical approaches that M_i_ is a stable monomer at low or high concentrations while M_s_ is predominantly a monomer at nanomolar concentrations but at micromolar concentrations, it exists as an equilibrium between a monomer and dimer. Finally, we used a structural approach to highlight changes in topology along the pathway to tau seed formation revealing structural rearrangements that involve the acidic N-terminus through the N1/N2 domains, the basic proline-rich domain (P1 and P2), and the repeat domain (RD). Our data support that brief incubation of tau with heparin following chromatographic separation produces a seed that is predominantly a monomer, which has the capacity to nucleate tau aggregation in vitro and in cells; and the monomeric seed displays distinct conformations. A deeper understanding of the conformation of tau seeds will yield insight into the mechanisms of structural polymorph formation and how ligands or cofactors can mediate this conversion into pathogenic forms of tau.

## Results and discussion

### Heparin-based conversion of tau into a pathogenic seed

**Figure 1:**
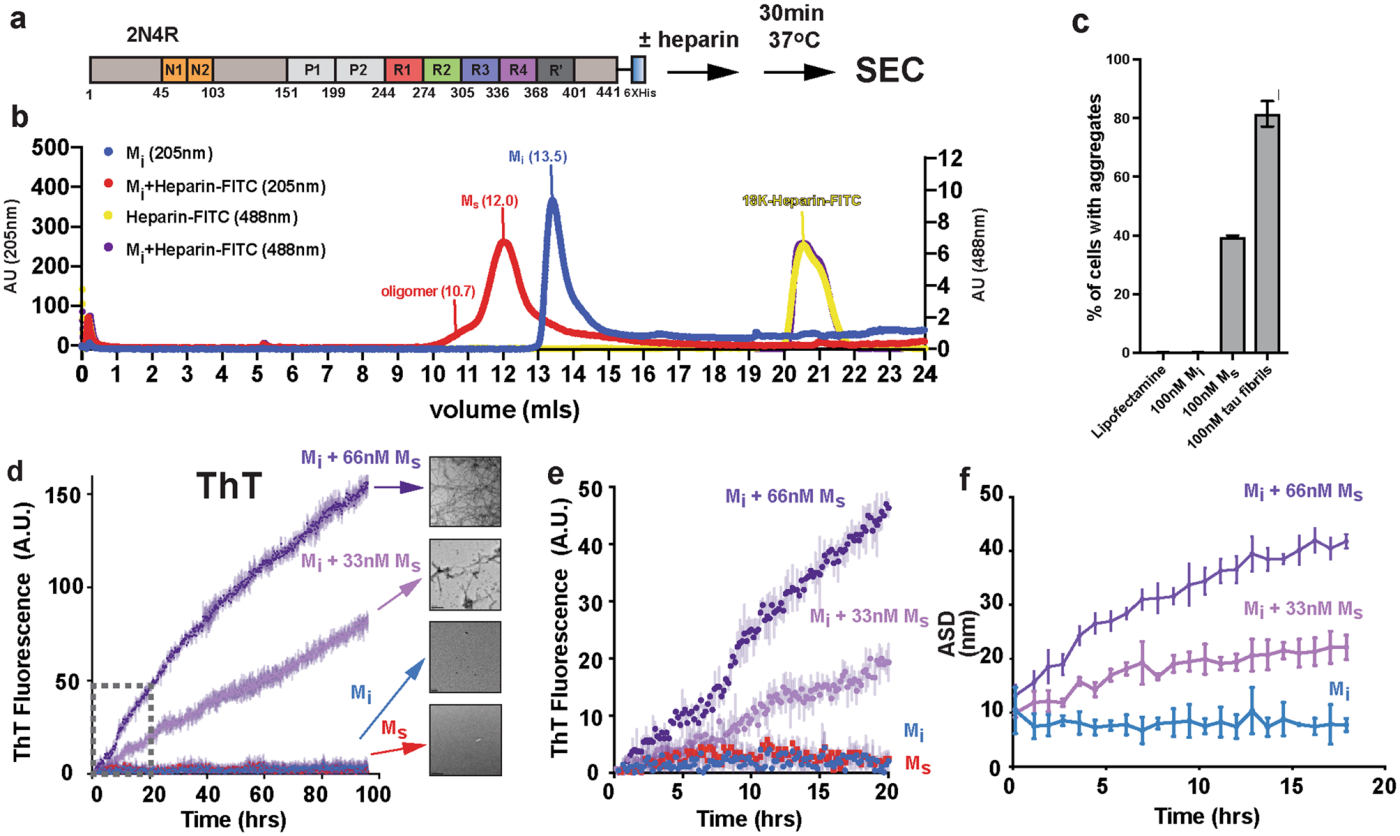
Generation and characterization of recombinant tau seeds. (**a**) Schematic for tau seed production. Full-length 2N4R tau was incubated with heparin for 30 min and resolved by SEC. 2N4R tau is shown as a cartoon schematic. The N1 and N2 domains are colored in orange. The repeat domains are colored in red, green, blue, magenta and dark grey. The proline-rich P1 and P2 domains are colored in light grey. (**b**) SEC chromatograph of tau, tau:heparin-FITC, heparin-FITC reactions resolved on a Superdex 200 10/300 GL increase column. The tau alone and tau:heparin-FITC traces acquired using Absorbance at 205nm are colored in blue and red, respectively. The heparin-FITC and tau:heparin-FITC traces acquired using Absorbance 488nm are colored in yellow and purple, respectively. (**c**) Activity of SEC fractions in a tau biosensor seeding assay. Lipofectamine alone and tau fibrils were used as negative and positive controls, respectively. Experiments were performed in triplicate showing average values with standard deviation. (**d**) ThT fluorescence aggregation assay comparing 4uM M_i_ (blue), 66 nM M_s_ alone (red), 4uM M_i_ + 33 nM M_s_ (light magenta), 4uM M_i_ + 66 nM M_s_ (purple). Representative TEM images of M_i_, M_s_, M_i_ + 33 nM M_s_ and M_i_ + 66 nM M_s_ samples imaged at the end point of each reaction. Fibrils were not observed in the M_i_ alone condition. Each experiment was performed in triplicate and is shown as an average with standard deviation. Grey dotted box highlights the data for the early 20 h time point for comparison with DLS data. (**e**) Zoom in of ThT fluorescence aggregation experiment from (**d**) within 20 h allowing direct comparison of the fluorescence signal to the DLS experiment in (**f**). Curves are colored as in (**d**). **(f)** DLS time-course of seeded M_i_ aggregation. Average size distribution (ASD) of triplicate 4uM M_i_ alone (blue), 4uM M_i_ + 33 nM M_s_ (light magenta) and 4uM M_i_ + 66n (purple). 66 nM M_s_ alone was not included because it was to dilute to observe by scattering. DLS experiment was carried out in triplicate and the data are shown as averages with standard deviation.

Initial efforts to produce small soluble tau seeds, including monomer, were based on sonication of tau fibrils followed by size exclusion chromatography (SEC) to separate the different sized species^4,18^. In vitro and in cell seeding assays confirmed the aggregation behavior of these small soluble species. Additionally, similarly sized species from different tauopathies were isolated from brain tissues^19,20^. Here we developed a method to consistently produce recombinant monomeric tau seeds without fibril sonication. Typically, tau aggregation involves incubation of tau in the presence of polyanions such as heparin in buffered saline. We discovered that incubation of these reactions in a sulfated buffer, such as 3-(N-morpholino) propanesulfonic acid (MOPS), slows the conversion of tau into larger oligomers allowing us to resolve small soluble species by SEC before they convert into large oligomers (Fig. 1a). We observed that on a Superdex 200 SEC column (GE), normal inert tau (herein M_i_) elutes as a monodisperse peak (Fig. 1b; blue trace, 13.5 ml) while tau incubated with heparin elutes earlier (Fig. 1b; red trace, 12 ml). To first evaluate the capacity of these tau species in mediating aggregation, we employed HEK293T tau biosensors that express tau repeat domain fused to CFP and YFP. This in-cell aggregation assay is sensitive, specific, and can detect tau aggregates down to femtomolar concentrations^21^. Transduction of negative (lipofectamine alone) and positive controls (recombinant tau fibrils) yielded expected signal with 0.14% ± 0.10 and 81.47% ± 4.32, respectively (Fig. 1c). Lipofectamine transduction of 100 nM M_i_ reisolated from SEC (i.e. Figure 1b, blue trace, 13.5 ml fraction) into biosensors cells yielded 0.17% ± 0.11 cells with aggregates (Fig. 1c), similar to lipofectamine alone. Transduction of 100 nM tau reisolated from the tau:heparin reaction (i.e. Figure 1b, red trace, 12 ml fraction) yielded 39.47% ± 0.55 of cells with aggregates (Fig. 1c). Given that the “seed” eluted earlier on an SEC column, we wondered whether this tau species is still bound to heparin. To monitor the heparin on the SEC, we utilized a heparin-fluorescein (FITC) conjugate allowing facile tracking of the absorbance signal at 488 nm. Heparin-FITC alone eluted at 20.5mls (Fig. 1b; yellow trace). The heparin-FITC absorbance for the tau:heparin-FITC reaction eluted as an overlapping peak with heparin-FITC alone (Fig. 1b; purple trace) with no detectible 488 nm signal in the fraction which induced tau aggregation in cells. To independently confirm the concentration of heparin-FITC signal in the M_s_ peak fraction (Fig. 1b, purple, 13mls), we produced a fluorescence calibration curve for FITC. We can detect FITC fluorescence signal down to 10e^-13^ M and it appears linear in the range from 0.05 nM to 1 μM (Supplementary Fig. S1a). Measurement of FITC fluorescence in the 13.5 ml peak from the tau:heparin reaction yielded an estimated 4 nM FITC signal while the A205 protein signal yields a tau concentration of 4 μM. This suggests that SEC is quite efficient at separating the heparin away from the tau leaving only trace amounts that are on the order of 1:1000 (Supplementary Fig. S1a). Our data support that brief incubation of recombinant tau with heparin followed by re-isolation by SEC produces small molecular-weight tau seeds that contain negligible amounts of heparin.

We wanted to show that our recombinant tau seeds are capable of inducing M_i_ aggregation using an in vitro Thioflavin T (ThT) fluorescence aggregation assay. Addition of 33 nM or 66 nM tau seeds to 4 μM M_i_ tau yields a robust increase in ThT fluorescence while M_i_ alone and M_s_ alone controls remained flat. We confirmed the presence of tau fibrils at the end of the reactions using Transmission Electron Microscopy (TEM). Consistent with the ThT experiments, inert tau samples treated with 33 nM or 66 nM seeds yielded fibrils by TEM while M_i_ or M_s_ alone did not (Fig. 1d,e). ThT fluorescence aggregation assays are the gold standard to detect β-sheet rich amyloid structures in vitro and we hypothesized that tau assembly can be monitored using dynamic light scattering (DLS). As in the ThT assay, M_i_ alone remained stable over the course of the 20 h experiment with an average size distribution (ASD) of 10 nm (Fig. 1f, Supplementary Data 1) skewed by a < 0.1% fraction of large species. Indeed, binning the data across sizes revealed predominantly a distribution of sizes ranging from 1 to 7 nm centered at 2.4 nm with no significant fraction of larger species (Supplementary Fig. S1b, Supplementary Data 1). Addition of 33 nM or 66 nM tau seeds to 4 μM M_i_ begins with an ASD around 10 nm (also skewed by < 0.1% larger species) and grows steadily over 20 h to 20 nm and 40 nm, respectively (Fig. 1f, Supplementary Data 1). Binning the sizes of these samples at t = 0 h showed similarity to the M_i_ alone condition, each seeded experiment predominantly starts as a distribution of sizes from 1 to 8 nm centered at 2.4 nm (Supplementary Fig. S1c,d, green, Supplementary Data 1). After 20 h in the 33 nM seeded samples, the smaller species shift to 4.2 nm and larger oligomers centered on 41.2 nm are now present (Supplementary Fig. S1c, red, Supplementary Data 1). After 20 h in the 66 nM seeded samples, the sizes shift yet further to species centered on 5.6 nm and 72.6 nm (Supplementary Fig. S1d, red, Supplementary Data 1). The DLS assay allows us to monitor the conversion of tau into larger species by following average sizes across the entire distribution but also to quantify the distribution of small and large oligomers over time. Interestingly, we can detect tau assembly using DLS at early time points where the ThT fluorescence is low and unreliable (Fig. 1e). Suggesting this is a good orthogonal assay to detect tau assembly early in aggregation reactions prior to robust ThT signal.

### Quantification of tau seed size

**Figure 2:**
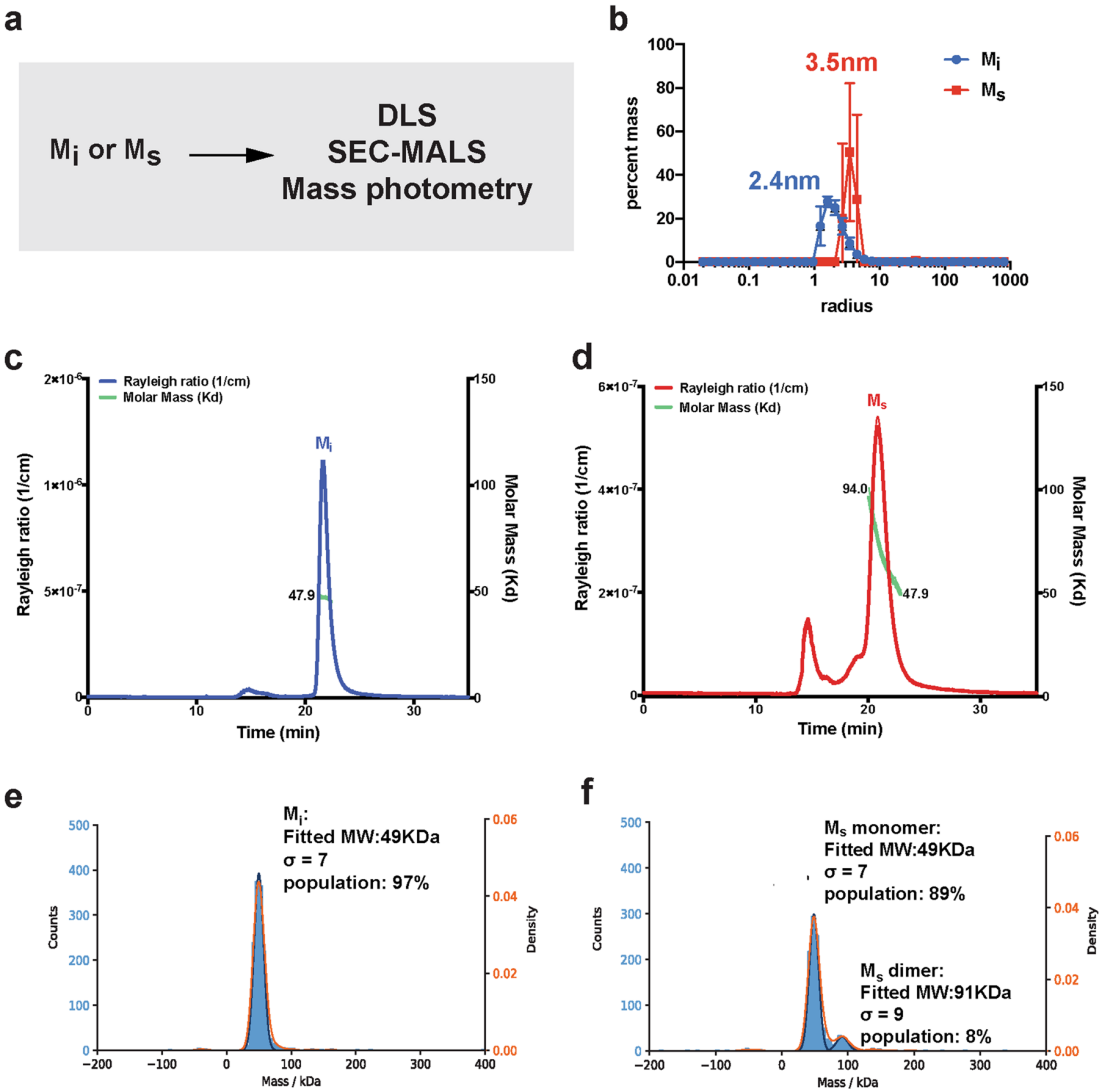
Quantification of tau seed shape and mass. (**a**) Schematic for experiments to measure the size of M_i_ and M_s_ using DLS, SEC-MALS and mass photometry. (**b**) Histogram of sizes observed for 4uM M_i_ (blue) and 4uM M_s_ (red) in DLS. (**c**) SEC-MALS of 16uM M_i_ (blue) shows a single peak that was calculated to have a molar mass of 47.9 g/mol (green). The single peak indicates that M_i_ tau elutes as a monomer. (**d**) SEC-MALS of 16uM M_s_ (red) shows a broader peak that was calculated to range from 94 g/mol to 47.9 g/mol (green). The broader peak indicates that M_s_ tau elutes as a distribution of dimer and monomer with minor signal from oligomers. (**e**) Mass photometry measurements of 50 nM M_i_ reveals the sample to be uniformly monomeric with a calculated molecular weight of 49 kDa accounting for 97% of the sample with a sigma of 7. (**f**) Mass photometry measurements of 50 nM M_s_ reveals the sample to be predominantly monomeric with a calculated molecular weight of 49 kDa accounting for 89% of the sample with a sigma of 7. We also observe a small proportion of a dimer with a calculated molecular weight of 91 kDa accounting for 8% of the sample with a sigma of 9.

To confirm the size of the heparin-induced tau seed species, we employed biophysical approaches including DLS, Size Exclusion Chromatography Multi-Angle Light Scattering (SEC-MALS) and mass photometry (Fig. 2a). First, we measured the size of M_i_ and the tau seed, M_s_, using DLS each at 4 μM. Our analysis suggests that the M_i_ size is small ranging in size between 1-7 nm and centered on 2.4 nm (Fig. 2b, blue, Supplementary Data 1) while the M_s_ tau seed has a narrower size distribution from 3 to 5 nm centered on 3.5 nm suggesting a larger size for the seed (Fig. 2b, red, Supplementary Data 1). While light scattering methods require high concentration samples, it is a method to unambiguously determine the molecular weight of protein species. Analysis of 44 μM M_i_ by SEC-MALS revealed good agreement with monomer with a molecular mass of 47.9 kDa (Fig. 2c). In contrast, analysis of 44 μM recombinant tau seeds induced by heparin revealed them to be a mixture of monomer (47.9 kDa) and dimer (94 kDa) with a minor population of oligomers eluting around the void volume (Fig. 2d). Capturing the true size of these seeds given their capacity to drive assembly under much lower concentration is challenging given the necessary concentration requirements (~ 2 mg/ml) for SEC-MALS. To circumvent this issue, we employed a mass photometry approach that utilizes the principles of interference reflection and scattering microscopy to measure molecular weight of samples in solution at dilute concentrations. We used this method to determine the molecular mass of our tau species using 100 nM concentrations, several orders of magnitude lower than required for SEC-MALS. We found that M_i_ is a monomer with a molecular weight of 49 kDa fitting 97% (s = 7) of the population (Fig. 2e). Analysis of the tau seeds showed that 83% of the population is 49 kDa (s = 7) with 11% of the population as a dimer with a molecular weight of 91 kDa (s = 9) (Fig. 2f). These analyses suggest that the seeds are indeed aggregation-prone and at high concentrations equilibrate between monomer and dimer while at low concentrations they remain predominantly monomer.

### Tau conformational changes in seed formation

**Figure 3:**
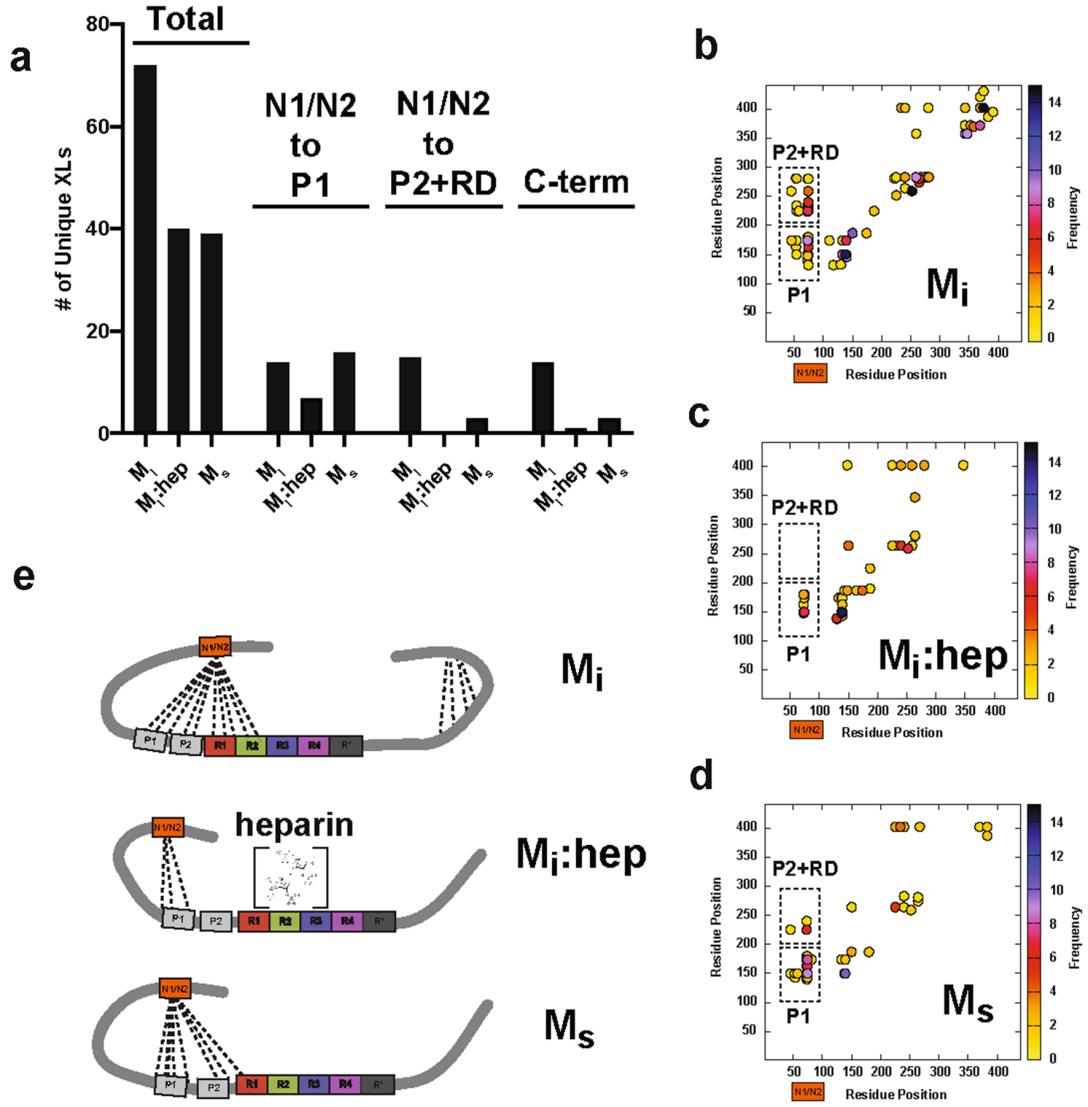
Topological changes in tau during seed formation. (**a**) Summary of unique crosslinks identified in M_i_, M_i_:heparin and M_s_. Experiments were performed as five replicates and data is shown as unique crosslinks observed across replicates. Total crosslinks are shown on the left, specific crosslinks observed from the acidic N-terminal N1/N2 domains (residues 45–101) to the P1 domain (residues 151–198, middle) and P2/RD domains (residues 199–368, right). Scatter plots of crosslinks identified in (**b**) M_i_, (**c**) M_i_:heparin and (**d**) M_s_ samples. Colors indicate the frequency of contacts. Dashed boxes highlight N-terminal N1/N2 contacts to P1 and P2/RD. (**e**) Model of tau conformational changes along the pathway of seed formation show changes in contacts from acidic N-terminal N1/N2 (orange) to P1/P2 (grey) and RD (red, green, blue and magenta).

Our prior studies on M_i_ and tau seeds involved crosslinking mass spectrometry (XL-MS) to understand possible changes in topology between these two conformations^3,4^. Our first experiments on tau employed a homo bi-functional disuccinimide suberate (DSS) crosslinker which reports on contacts between lysine residues^22^. We have recently employed a new chemistry that can report on zero-length crosslinks directly between lysine residues and amino acids containing carboxylic acids mediated by 4-(4,6-Dimethoxy-1,3,5-triazin-2-yl)-4-methylmorpholinium chloride (DMTMM). In full-length tau we observed contacts between the acidic N-terminus and the basic repeat domain and we showed that pathogenic mutations including P301L alter the distribution of contacts from the N-term to the repeat domain^22^. Building on these ideas, we wondered how the N-term contacts to the repeat domain in full-length tau change from M_i_ to a seed. We reacted Mi, M_i_:heparin and M_s_ with DMTMM for 15 min, the samples were resolved by SDS-PAGE (Supplementary Fig. 2a-c) and the monomer bands were extracted from the gel. The samples were processed using our XL-MS pipeline to identify changes in crosslink contacts with regard to XL sites and frequencies in these three different states. We observe 72 crosslinks in M_i_ (Fig. 3a, Supplementary Data 2) compared to 40 and 39 for M_i_:heparin and M_s_, respectively (Fig. 3a, Supplementary Data 2). Additionally, we find that the M_i_ contacts are more heterogeneous, which are consistent with tau being an intrinsically disordered protein. Meanwhile, the M_i_:heparin and M_s_ contacts exhibit more reproducible modes of contacts (Supplementary Fig. S2d–f). Consistent with our previous observations in full-length 2N4R tau^22^, in M_i_ we observe 14 and 15 contacts from the N-terminal acidic N1/N2 domains to Proline-rich Domain 1 (P1) and Proline-rich Domain 2 (P2)/RD, respectively (Fig. 3a,b). In the M_i_:heparin complex, we observe 7 contacts from the N-term N1/N2 to P1 but a complete loss of contacts from the N-term N1/N2 to P2/RD (Fig. 3a,c). In M_s_, which is repurified from heparin incubation using SEC we recover 15 contacts from the N-term N1/N2 to P1 (Fig. 3a,d) but only 3 contacts from the N-term to P2/RD are recovered (Fig. 3a,d). Notably, we also observe a loss of crosslinks in the C-terminus in M_i_:heparin and M_s_ relative to M_i_ (Fig. 3b–d). These data indicate that in the M_i_:heparin complex, the polyanion binds to the RD and displaces contacts from the acidic N-term N1/N2 to basic P1/P2 and RD. In M_s_ conformation, where the heparin has been removed, leaves the RD more exposed. In addition to crosslinks, our XL-MS data also yield information about solvent accessibility by monitoring the crosslinker reactions to a single amino acid which can be used as a measure of solvent accessibility. Consistent with the crosslinking data, we observe a higher incidence of adipic acid dihydrazide (ADH) monolinks (i.e. solvent exposure) at the acidic N-term N1/N2 in the M_i_:heparin complex compared to the M_i_ and M_s_ samples (Supplementary Fig. S2g–i). Importantly, we have previously proposed that local protective structures engage amyloid motifs to prevent their self-assembly and consistent with this model heparin may play a role in disrupting both local structure and displacing long-range aggregation-protective contacts from the acidic N-term N1/N2 to P1 and P2/RD.

### Implications for tau aggregation

The structural polymorphisms of tau fibrils are each linked to distinct tauopathies. Thus reagents, such as antibodies or small molecules, specific for different fibrillar structures could be used to accurately diagnose late-stage diseases. Our prior work indicated that small soluble species, including monomer, isolated from either recombinant or tauopathy tissue sources can drive aggregation of inert tau and mediate self-assembly^4^. Structural analysis of recombinant seeds and AD-derived seeds suggested some conserved contacts in their overall topology^4^. More recently, work from Sharma et al.^15^ revealed that small seeds isolated from disease encode conformational plasticity to maintain a subset of structural polymorphs. It remains unclear how tau seeds form and drive formation of structural polymorphs in disease but it is likely that ligand binding or specific post-translational modifications (PTMs) may promote seed formation to initiate disease. While heparin-induced seeds are likely not physiological, other polyanions such as RNA, may be an important ligand that initiates this process^23,24^. Our data support that heparin binding to the repeat domain of tau displaces specific long-range contacts to the acidic N-terminal N1/N2 domains. Subsequent chromatographic repurification of tau:heparin complexes strips the heparin away allowing the recovery of N-term N1/N2 to P1 contacts but the P2 and RD surfaces remain unmasked which could expose putative amyloid motifs thus promote self-assembly. This model may explain the role of cofactors or PTMs in unmasking specific surfaces for subsequent assembly. It also reveals a role of N-terminal N1/N2 contacts to the proline-rich domain to stabilize monomeric forms of seeds. Additionally, for many years post-translational modifications, such as phosphorylation, were proposed to drive this process^25,26^ but direct evidence for a role of phosphorylation on tau remains unclear. Cryo-Electron Microscopy (cryo-EM) structures of tau fibrils isolated from tauopathy brain tissues suggested the role of acetylation and ubiquitination in the formation of corticobasal degeneration (CBD) and Alzheimer’s disease (AD) structural polymorphs^27,28^ but whether this initiates the process or accumulates once these structures are formed remains an open question. Moreover, disease-derived cryo-EM structures of tau have revealed the potential role of yet unknown ligands highlighted by unexplained density in the fibril structures^27,29^. How seeds relate to structural polymorphs of tau remains elusive but specific exposure of amyloid motifs or ordering of interactions between motifs is likely a central process to mediating tau aggregation. It is also interesting to consider why under non-disease conditions tau does not aggregate in cells given that polyanions, such as RNA, are present in cells. We suspect that under these conditions, in addition to local protective structures surrounding the amyloid motifs^3,4^, molecular chaperones may play a role in limiting tau seed formation while in disease states these processes are dysregulated^22^. A deeper understanding for how pathogenic seeds are formed and how they drive the formation of distinct structural polymorphs will be required to understand the mechanism of tauopathies and reveal new insight into their treatment.

## Materials and methods

### Tau purification and SEC of tau species

Wild-type 2N4R tau in PET28b plasmid with a C-terminal 6 × His tag was expressed in *E. Coli* BL21 (DE3) and purified using the same protocol as previously described^3^. The production of tau seeds was carried out by incubating 16uM wild-type tau with 1:1 molar ratio of heparin (AMSbio, AMS.LMW Heparin) or heparin-FITC (Creative PEGWorks, HP-201, 18 kDa) for 1 h at 37 °C without shaking in 30 mM MOPS pH7.4, 50 mM KCl, 5 mM MgCl_2_ and 1 mM with DTT (MOPS buffer). The tau:heparin reactions were injected immediately onto a Superdex 200 Increase 10/300 GL (GE) equilibrated in 1xPBS (sigma 45ZP17) yielding a peak that eluted around 1mls earlier than untreated tau. The seeding activity was confirmed using tau FRET biosensor cells (see below). The bump/dip variation around the bed column volume was normalized to the same buffer. To quantify the remaining heparin in eluted M_s_, heparin Fluorescein (Creative PEGWorks, HP-201 18 kDa) alone and in complex with 1:1 molar ratio of 16uM tau were both injected on SEC. The fluorescence intensity from the eluted M_s_ peak in 1xPBS (sigma 45ZP17) was measured on a Tecan infinite M1000 plate reader. The standard calibration curve was prepared using the same heparin-FITC in 1xPBS (sigma 45ZP17) and also measured on a plate reader.

### Tau seeding

Samples from SEC elutions were assayed for their seeding activity in HEK293T tau biosensor cells and compared to the same amount of heparin-induced recombinant tau fibril^30^. For all experiments, cells were plated in 96-well plates at 20,000 cells per well in 100 µl of media. 24 h later, the cells were treated with 30 µl sample:lipofectamine complex. Prior to cell treatment, the recombinant tau fibrils were sonicated for 30 s at an amplitude of 65 on a Q700 Sonicator (QSonica). 48 h after treatment with tau, the cells were harvested by 0.05% trypsin digestion and then fixed in 1xPBS (sigma 45ZP17) with 2% paraformaldehyde. A BD LSRFortessa was used to perform FRET flow cytometry. To measure mCerulean and FRET signal, cells were excited with the 405 nm laser and fluorescence was captured with a 405/50 nm and 525/50 nm filter, respectively. To measure mClover signal, cells were excited with a 488 laser and fluorescence was captured with a 525/50 nm filter. To quantify FRET, we used a gating strategy where mCerulean bleed-through into the mClover and FRET channels was compensated using FlowJo analysis software. As described previously^3^. FRET signal is defined as the percentage of FRET-positive cells in all analyses. For each experiment, 10,000 cells per replicate were analyzed and each condition was analyzed in triplicate. Data analysis was performed using FlowJo v10 software (Treestar).

### ThT aggregation assay

Wild-type 2N4R tau was diluted to 17.6 µM in MOPS buffer with 25 mM β-mercaptoethanol and boiled at 100 °C for 5 min. The boiled tau samples were first diluted two-fold with 1xPBS (sigma 45ZP17) to 8.8uM tau and a final concentration of 25 µM ThT was added in dark. For a 60 µl reaction system, 30 µl diluted boiled tau:ThT was mixed with equal volume of a mixture consisting of either buffer, seeding monomer with final concentration at 33 nM or 66 nM or any combination of them^3^. All experiments were performed in triplicate. ThT kinetic scans were run every 10 min on a Tecan Spark plate reader at 446 nm Ex (5 nm bandwidth), 482 nm Em (5 nm bandwidth) with agitation for 5 s prior to acquisition.

### Transmission electron microscopy

The TEM experiments were performed similar to as described previously^3^. Briefly, an aliquot of 5 μl sample was loaded onto a glow-discharged Formvar-coated 300-mesh copper grids for 30 s and was blotted by filter paper followed by washing the grid with 5 μl ddH_2_O. After another 30 s, 2% uranyl acetate was loaded on the grids and blotted again. The grid was dried for 1 min and loaded into a FEI Tecnai G2 Spirit Biotwin TEM. All images were captured using a Gatan 2Kx2K multiport readout post column CCD at the UT Southwestern EM Core Facility.

### Dynamic light scattering aggregation assay

The DLS experiments were performed similar to as described previously^31^. Reactions were prepared using the same experimental conditions as the ThT fluorescence aggregation experiments. Briefly, wild-type 2N4R tau was diluted to 17.6 µM in MOPS buffer with 25 mM β-mercaptoethanol and boiled at 100 °C for 5 min. The boiled tau samples were first diluted two-fold in 1xPBS (sigma 45ZP17). For a 60 µl reaction volume, 30 µl diluted boiled tau protein was mixed with equal volume of a mixture consisting of either buffer or seeding monomer (final concentration with 33 nM or 66 nM). All protein samples were filtered through a 0.22 μm PES sterile filter and loaded in triplicate onto a 384 well clear flat-bottom plate. The plate was loaded into a Wyatt DynaPro Plate Reader III and set to run continuously at room temperature at a scanning rate of 1 scan per 15 min, with 1 scan composed of 10 acquisitions for 18 h. The data were analyzed using the Wyatt Dynamics software version 7.8.2.18. Light scattering results were filtered by Sum of Squares (SOS) < 20 to eliminate statistical outlier acquisitions within each scan. Data were reported as average R_h_ over the time course.

### SEC-MALS

The SEC-MALS experiments were performed similar to as described previously^31^. 2 mg/ml of M_i_ alone and in complex with 1:1 molar ratio of heparin (AMSbio, AMS.LMW Heparin) were filtered through a 0.22 μm PES filter before 100 μL each was applied to a Superdex 200 Increase 10/300 column (GE) equilibrated in 1xPBS (sigma 45ZP17) with 1 mM TCEP. The column was in line with a Shimadzu UV detector, a Wyatt TREOS II light-scattering detector, and a Wyatt Optilab tREX differential-refractive-index detector. The flow rate was 0.5 ml/min. The data were analyzed with Wyatt’s ASTRA software version 7.1.0.29. SEDFIT was used to calculate the dn/dc of the protein.

### Mass photometry

M_i_ and M_s_ data were acquired in Refeyn OneMP mass photometer. Prior to the measurement, 50 uM tau M_i_ was diluted 900X times to 55.5 nM and 200 nM M_s_ was diluted 30X times to 6.6 nM in 1xPBS (sigma 45ZP17). 15 µl of each diluted tau sample was injected into the flow-chamber and movies of either 60 or 90 s duration were recorded after autofocus stabilization. Data was processed by Refeyn team through Gaussian fitting to provide the peak mass, the sigma (standard deviation) of the Gaussian, and the number of particles under that Gaussian (and as a % of all counts in the graph). Percentages are with respect to all the particles contained in the graph. Overlapping Gaussian curves will over-count the number of particles in a given population.

### XL-MS of different tau samples

We have developed standardized protocols for crosslinking and data analysis of samples. For DMTMM reactions, protein samples were crosslinked at 0.3 mg/ml in 100 µl total volume with a final 12 mg/ml DMTMM for 15 min at 37 °C while shaking at 750 rpm. For ADH/DMTMM reactions, protein samples were crosslinked at 0.3 mg/ml in 100 µl total volume with a final 8.3 mg/ml ADH (d_0_/d_8_, Creative Molecules) and 12 mg/ml DMTMM (Sigma-Aldrich) for 15 min at 37ºC while shaking at 750 rpm. The reactions were quenched with 200 mM Ammonium Bicarbonate (AB) for 30 min. Samples were resolved on SDS-PAGE gels (NUPAGE™, 4 to 12%, Bis–tris, 1.5 mm or home-made SDS-Gel) and bands corresponding to tau monomer were gel-extracted following standard protocols^3^. Samples were flash frozen in liquid nitrogen, lyophilized and resuspended in 8 M urea followed by 2.5 mM TCEP reduction and 5 mM Iodoacetamide alkylation in dark with each 30 min. Samples were then diluted to 1 M urea by 50 mM AB and digested by 1:50 (m/m) trypsin (Promega) overnight shaking at 600 rpm. 2% (v/v) formic acid was added to acidify the reaction system and further purified by reverse-phase Sep-Pak tC18 cartridges (Waters) and size exclusion peptide chromatography (SEPC). Fraction collected from SEPC was lyophilized. The dried samples were resuspended in water/acetonitrile/formic acid (95:5:0.1, v/v/v) to a final concentration of approximately 0.5 µg/µl. 2 µl of each was injected into Eksigent 1D-NanoLC-Ultra HPLC system coupled to a Thermo Orbitrap Fusion Tribrid system at the UTSW Proteomics core.

The analysis of the mass spectrum data was done by in-house version of xQuest^32^. Each Thermo.raw data was first converted to open.mzXML format using mscovert (proteowizard.sourceforge.net). Search parameters were set differently based on the crosslink reagent as followed. For DMTMM zero-length crosslink search: maximum number of missed cleavages = 2, peptide length = 5–50 residues, fixed modifications = carbamidomethyl-Cys (mass shift = 57.02146 Da), mass shift of crosslinker = − 18.010595 Da, no monolink mass specified, MS1 tolerance = 15 ppm, and MS2 tolerance = 0.2 Da for common ions and 0.3 Da for crosslink ions; search in enumeration mode. For ADH, maximum number of missed cleavages (excluding the crosslinking site) = 2, peptide length = 5–50 residues, fixed modifications = carbamidomethyl-Cys (mass shift = 57.021460 Da), mass shift of the light crosslinker = 138.09055 Da, mass shift of monolinks = 156.10111 Da, MS1 tolerance = 15 ppm, MS2 tolerance = 0.2 Da for common ions and 0.3 Da for crosslink ions, search in ion-tag mode. FDRs were estimated by xprophet^33^ to be 0–0.17%. For ADH For each experiment, five replicate data sets were compared and the frequency of contacts were calculated. The pairs position and unique nseen numbers (frequencies) were visualized using custom gunplot script.

## Supplementary Information

Supplementary Data 1.

Supplementary Data 2.

Supplementary Figures.

## Data Availability

Raw DLS data is available in Supplemental Data 1. Raw crosslinking mass spectrometry data is available in Supplemental Data 2. Other data sets generated during and/or analyzed during the current study are available from the corresponding author on reasonable request.
