# Supplementary material for: Biophysical properties of a tau seed
Source: Sci Rep. 2021 Jun 30;11:13602. doi: 10.1038/s41598-021-93093-z (PMC8245522; doi:10.1038/s41598-021-93093-z)
Supplement: Supplementary file 3 — Supplementary Figures. [file 41598_2021_93093_MOESM3_ESM.docx]

SUPPLEMENTARY INFORMATION

**Biophysical properties of seed-competent tau monomer**

Zhiqiang Hou^1^, Dailu Chen^1,2^, Bryan D. Ryder^1,2^, Lukasz A. Joachimiak^1,3*^

SUPPLEMENTARY FIGURES AND LEGENDS

**
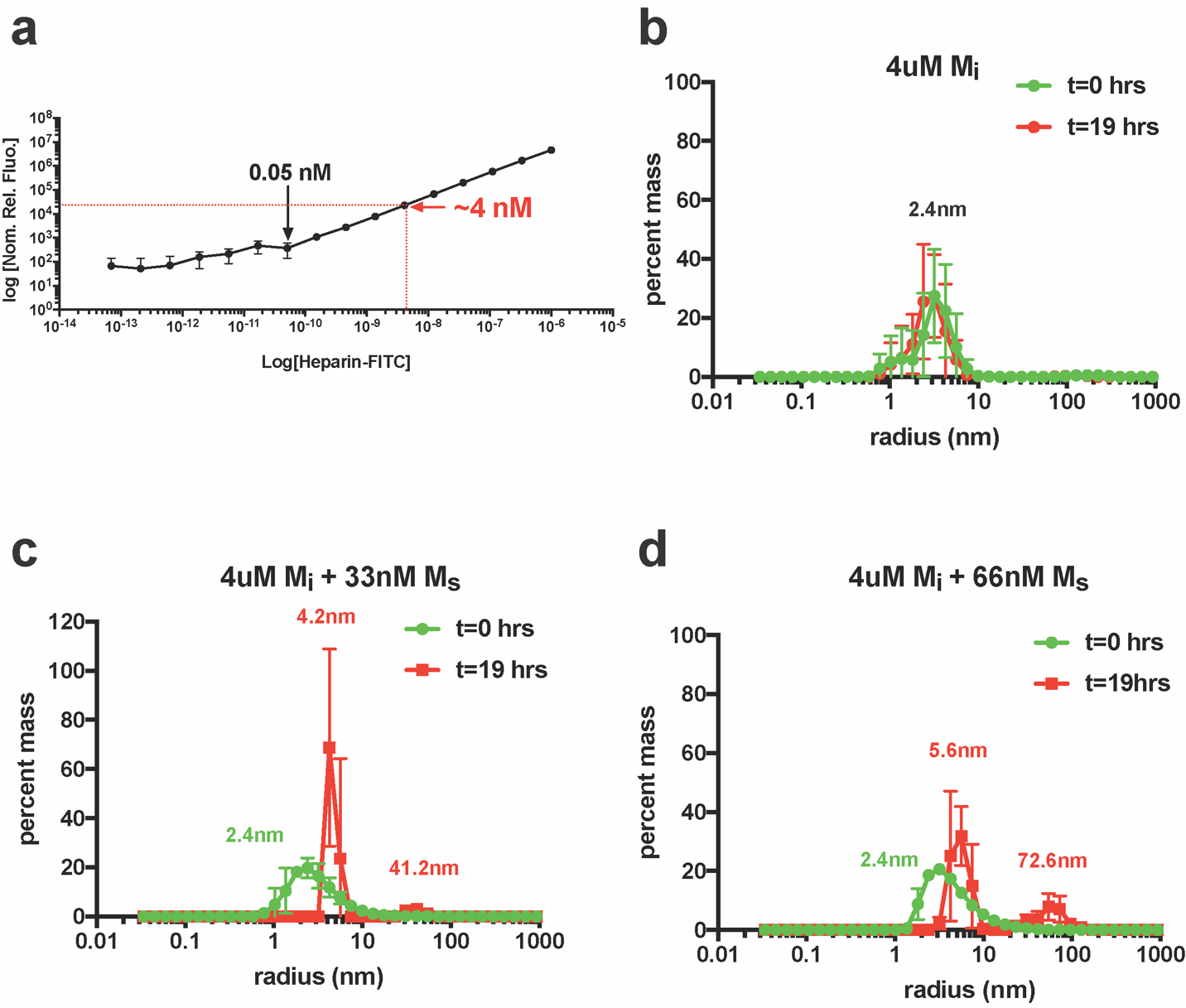
Supplementary Figure 1. Assembly properties of a tau seed. (a)** Calibration curve of FITC fluorescence using excitation 495nm and emission 520nm. Signal detected in the M_i_:heparin-FITC reaction from the M_s_ fraction corresponds to 4 nM FITC. **(b)** Histogram of size distributions of 4uM M_i_ at times 0 hour (green) and 19 hour (red). Median size is indicated above the distribution. **(c)** Histogram of size distributions of 4uM M_i_ with 33 nM M_s_ at times 0 hour (green) and 19 hour (red). **(d)** Histogram of size distributions of 4uM M_i_ with 66 nM M_s_ at times 0 hour (green) and 19 hour (red). Median species size is indicated above the distribution and is colored green for 0 hour and red for 19 hour. Each DLS experiment was performed in triplicate and the data is shown as averages with standard deviation.

**
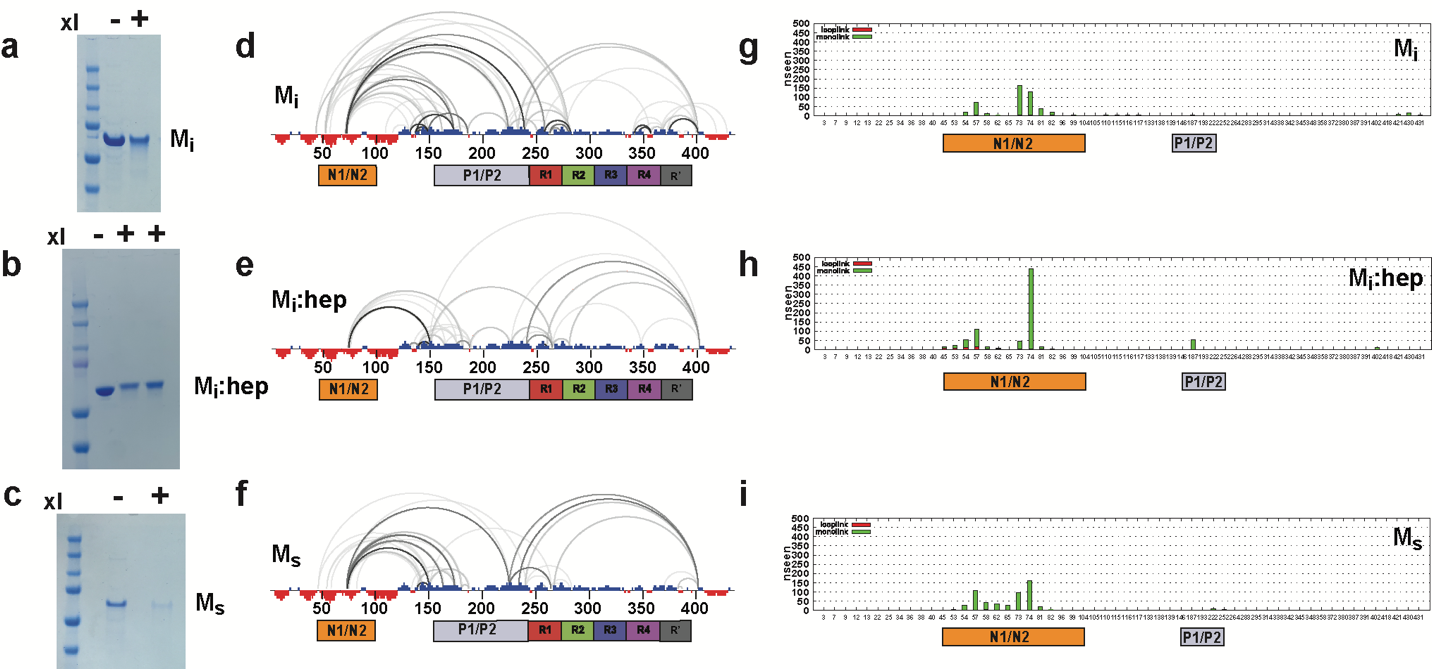
**

**Supplementary Figure 2. (a-c)** M_i_, M_i_:heparin and M_s_ samples were reacted with DMTMM/ADH for 15 minutes and the non-crosslinked and crosslinked reactions were resolved on SDS-PAGE. Linear plots illustrating the distribution of zero-length contacts in M_i_ (**d**), M_i_:heparin (**e**) and M_s_ (**f**) across 5 replicate samples. The crosslinks are shown as semi-circles and are colored according to frequency in the experiments. Cartoon schematic for 2N4R tau highlighting the N1/N2, P1/P2 and repeat domains colored as in Fig 1a. (**g-i)** Frequencies of monolinks (green) and looplinks (red) derived from the M_i_, M_i_:heparin and M_s_ crosslinking reactions. Modifications are derived from ADH reactions and are shown as a bar plot using the mean frequency of monolinks and looplinks across 5 replicates. ADH monolinks and looplinks are predominantly observed in the acidic N-terminal N1/N2 (orange) and their relative frequency can be interpreted as solvent accessibility. Only minor changes are observed in the P1/P2 region (grey).
